# Supplementary material for: Interstitial Lung Disease in Immunocompromised Children
Source: Diagnostics (Basel). 2022 Dec 26;13(1):64. doi: 10.3390/diagnostics13010064 (PMC9818431; doi:10.3390/diagnostics13010064)
Supplement: Supplementary file 1 [file diagnostics-13-00064-s001.zip › diagnostics-2080301-supplementary.pdf]

Supplemental Table S1. Immunodeficiency groups and diagnosis

Disease group (n)	Diseases (n)
Primary immuno-deficiencies (120)	
Combined deficiencies (22)	AR-CD40 def.CD40 (2), AR-ADA def.ADA (2), AR-DOCK8 def.DOCK8 (1), AR-MHC-II GrD def.RFXAP (1), XL-CD40 ligand def.CD40LG (1), XL-CD132 def.IL2RG (1), CID-MHC-II def (1), SCID (7), Combined ID not further differentiated (6)
Well-defined syndromes (17)	Di George/velocardiofacial Syndrome (4), AR-Ataxia-telangiectasia.ATM (3), Chromosome 10p13-p14 deletion syndrome.10p13-p14 (1), AD-EDA-ID due to IKBA GOF mutation.IKBA (1), AR-ICF1.DNMT3B (1), AR-ID with multiple intestinal atresias.TTC7A (1), AR-MCM4 def.MCM4 (1), AR-Nijmegen breakage syndrome.NBS1 (1), HELLS (1), CID_syndromic not further differentiated (1)
Antibody deficiencies (21)	AD-NFKB1 def.NFKB1 (1), AD-PIK3CD mutation (GOF).PIK3CD GOF (1), TACI def.TNFRSF13B (1), XLA.BTK (1), Antibody deficiency (8), IgA deficiency (4), Isolated IgG subclass def (3), Transient hypogamma infancy (1), CVID (1)
Immune dysregulation (5)	XL-IPEX.FOXP3 (2), AR-IL-10 def.IL10 (1), AD-STAT3 GOF mutation.STAT3 (1), AR-UNC13D Munc13-4 def (FHL3).UNC13D (1)
Defects of phagocytes (30)	AR-CGD.CYBA (3), XL-CGD.CYBB (1), AR-CGD.NCF2 (1), AR-CGD.NCF4 (1), XL-PAP.CSF2RA (15), Shwachman-Diamond syndrome (2), Neutropenia with combined immune def (3), CGD not further differentiated (4)
Defects of innate immunity (7)	AR-ZNFX-1 def.ZNFX-1 (4), AR-MDA5 def (LOF).IFIH1 (1), AD-STAT1 def.(LOF).STAT1 (1), AR-TCIRG1 def.osteopetrosis.TCIRG1 (1)
Autoinflammatory syndromes (17)	AD-COPA def.COPA (7), AD/AR-FMF.MEFV (2), AD-TRAPS.TNFRSF1A (2), AD-OAS1 deficiency .OAS1 GOF (1), AD-AGS7.IFIH1 GOF (1), AD-PLAID , or APLAID.PLCG2 GOF (1), AR-STING-associated vasculopathy, infantile-onset.TMEM173 (1), STING-associated vasculopathy, infantile-onset (1), Autoinflammatory disorder (1)
Bone marrow failure (1)	AD/AR-DKC def.TERT (1), AD-DKC def.TERC (1), AD-MIRAGE.SAMD9 GOF (1)
Secondary immuno-deficiencies (97)	ALL (15), AML (10), CLL (2), CML (1), HIV (2), Hodgkin lymphoma (3), JMML (3), MDS (5), Non Hodgkin lymphoma (1), Cancer not further differentiated (10), Other therap. intervention (1), Transplant-heart (3), Transplant-heart and lung (6), Transplant-kidney (1), Transplant-lung (4), Transplant-stem cell (30)

Supplemental Table S2. Definition of final lung diseases diagnosis

Term used in current publication for the group of lung diseases indicated in the left column	Lung diseases and diagnosis retrieved form the patients` records and summarized under the term in the left column
Opportunistic/recurrent infection	Pneumonia, purulent tracheobronchitis, pulmonale aspergillosis, bronchopneumonia, bronchitis, recurrent bronchitis, (chronic) pulmonary infiltrate, recurrent pneumonia, pleuropneumonia bilateral, recurrent airway infections, recurrent nocturnal cough and dyspnea under stress, purulent hemorrhagic tracheobronchitis, recurrent fever obstructive bronchitis, pneumatoceles, multifocal bronchomalacia, dystelectatic abscessing pneumonia, fibrinous purulent pneumonia, residual lung calcifications after pneumocystis, atelectasis, pneumocystis pneumonia, lung infection, recurrent respiratory infections, aspergilloma with foci of both lungs, suppurative lung disease, severe post-transplantation lung disease Epstein-Barr virus reactivation
Bronchiolitis obliterans (BO)	Bronchiolitis obliterans, pulmonary graft versus host disease
Asthma	Wheezing, bronchial asthma, obstructive lung disease, chronic obstructive pulmonary disease (COPD), peripheral obstruction of the lung, bronchial hyperreactivity, bronchial hyperresponsiveness, recurrent bronchial obstruction, chronic steroid-dependent lung disease, oppressed breathing, allergic bronchopulmonary aspergillosis, recurrent obstructive bronchitis
Bronchiectasis	Bronchiectasis, middle lobe syndrome
Interstitial lung disease (ILD)	Granulomatous pneumonitis, interstitial pneumonia, lung fibrosis, pulmonary fibrosis, lymphoid granulomatosis, bronchocentric granulomatosis, pleuropulmonary elastosis, granulomatous pneumonitis, Desquamative interstitial pneumonia (DIP), Bronchopulmonary dysplasia (BPD), Pulmonary Alveolar Proteinosis (PAP), pleuroparenchymal fibroelastosis, focal intra alveolar fibrosis, Lymphocytic interstitial pneumonitis (LIP), cholesterol pneumonia, Granulomatous and Lymphocytic Interstitial Lung Diseases (GLILD), Nonspecific interstitial pneumonia (NSIP), lymphofollicular bronchiolitis, restrictive lung disease, interstitial thickening, clear fibrosis, bronchocentric granulomatosis, connective tissue disease with lung involvement, cicatricial fibrosis, alveo-interstitial lung disease, pulmonary hemosiderosis, hemorrhagic pneumonitis, multiple hemorrhagic pulmonary edema, pulmonary hemorrhage
ARDS	Acute respiratory distress syndrome (ARDS), Respiratory distress syndrome (RDS)
Respiratory failure (RF)	Respiratory insufficiency, tachydyspnea, lung failure, respiratory failure, respiratory decompensation, respiratory partial failure
Pneumothorax	Recurrent pneumothorax, pneumothorax, spontaneous pneumothorax

Pulmonary hypertension (PHT)	Portopulmonary hypertension (PPHT), pulmonary hypertension, stenosis of the pulmonary artery, pulmonary stenosis, dilation of the pulmonary artery, pronounced obliterative vasculopathy
Diffuse alveolar wall damage (DAD)	Diffuse alveolar damage (DAD)
Pleural disease	Subpleural fibrosis, pleural fibrosis, pleurisy, pleural effusion, pleural empyema
Post-transplant lymphoproliferative disorders (PTLD)	PTLD
